# Influx of Backyard Farming with Limited Biosecurity Due to the COVID-19 Pandemic Carries an Increased Risk of Zoonotic Spillover in Cambodia

**DOI:** 10.1128/spectrum.04207-22

**Published:** 2022-12-14

**Authors:** Sudipta Hyder, Benjamin L. Sievers, Claude Flamand, Damian TagoPacheco, Malen Chan, Filip Claes, Erik A. Karlsson

**Affiliations:** a Virology Unit, Institut Pasteur du Cambodge, Phnom Penh, Cambodia; b Epidemiology and Public Health Unit, Institut Pasteur du Cambodge, Phnom Penh, Cambodia; c Emergency Center for Transboundary Animal Diseases (ECTAD) of the Food and Agriculture Organization of the United Nations, Regional Office for Asia and the Pacific, Bangkok, Thailand; d CANARIES, Consortium of Animal Networks To Assess Risk of Emerging Infectious Diseases through Enhanced Surveillance, Institut Pasteur du Cambodge, Phnom Penh, Cambodia; University of Georgia

**Keywords:** COVID-19, food insecurity, poultry raising, avian influenza virus, antimicrobial resistance, tour guide, poultry farmer, backyard farming, biosecurity, poultry, zoonoses

## Abstract

Backyard farming with limited biosecurity creates a massive potential for zoonotic spillover. Cambodia, a developing nation in Southeast Asia, is a hub for emerging and endemic infectious diseases. Due to pandemic-induced job losses in the tourism sector, rumors suggest that many former Cambodian tour guides have turned to backyard farming as a source of income and food security. A cross-sectional study including 331 tour guides and 69 poultry farmers in Cambodia before and during the novel coronavirus disease 2019 (COVID-19) pandemic was conducted. Participants were administered a survey to assess food security, income, and general farming practices. Survey data were collected to evaluate the risk perceptions for avian influenza virus (AIV), antimicrobial resistance (AMR), and general biosecurity management implemented on these poultry farms. Overall, food security decreased for 80.1% of the tour guides during the COVID-19 pandemic. Approximately 21% of the tour guides interviewed used backyard poultry farming to supplement losses of income and food insecurity during the COVID-19 pandemic, with a significantly higher risk than for traditional poultry farmers. Agricultural intensification in Cambodia due to the COVID-19 pandemic has caused an influx of makeshift farms with limited biosecurity. Inadequate biosecurity measures in animal farms can facilitate spillover and contribute to future pandemics. Improved biosecurity and robust viral surveillance systems are critical for reducing the risk of spillover from backyard farms.

**IMPORTANCE** While this study highlights COVID-19-associated changes in poultry production at a small scale in Cambodia, poultry production is expected to expand due to an increase in the global demand for poultry protein during the pandemic, changes in urbanization, and the reduction of the global pork supply caused by African swine fever (ASF). The global demand and surge in poultry products, combined with inadequate biosecurity methods, can lead to an increased risk of domestic animal and human spillovers of zoonotic pathogens such as avian influenza. Countries in regions of endemicity are often plagued by complex emergency situations (i.e., food insecurity and economic fallouts) that hinder efforts to effectively address the emergence (or reemergence) of zoonotic diseases. Thus, novel surveillance strategies for endemic and emerging infectious diseases require robust surveillance systems and biosecurity training programs to prevent future global pandemics.

## INTRODUCTION

Aside from massive impacts on public health, the coronavirus disease 2019 (COVID-19) pandemic significantly affected the global workforce, especially in the tourism and hospitality industries. Before the pandemic, tourism supported 1 in 10 jobs and provided a livelihood for millions more in both developing and developed economies (1). Cambodia, a least developed country in the Greater Mekong Subregion of Southeast Asia, was no exception to this global industry layoff. Travel and tourism accounted for 32.7% of Cambodia’s gross domestic product (GDP) in 2019 (this year also recorded the highest volume of inbound travelers) (2). The decline in inbound tourists has been worsened by the travel bans imposed in Cambodia during the pandemic. Foreign tourist arrivals declined from 6,610,592 (2019) to 1,306,143 (2020), reaching only 196,495 visitors by 2021 (3). Once a thriving hub attracting millions of visitors annually, Siem Reap has been devastated by COVID-19; approximately 60 to 70% of individuals employed in tourism have lost their jobs or have been temporarily suspended from work (3).

Job loss leads to instability in the most fundamental categories of Maslow’s hierarchy: physiological needs for food, drink, and shelter (4). While the cultivation of food and goods through smallholder farming produces the vast majority of the world’s food supply and economy (5), it also serves to supplement food scarcity and income in times of hardship. Indeed, during times of economic crisis and food scarcity, such as economic fallout and war, intensified backyard practices have occurred sporadically, such as vacant-lot gardens (6), thrift gardens (7), and victory gardens (8), to name a few. Amid the COVID-19 pandemic, backyard and community farming practices have also surged in popularity (9) as they present an attractive alternative to an industrial food system wracked with supply issues and are a relatively simple way to achieve food security and income (10).

In addition to tourism, Cambodia is also highly dependent on agriculture and livestock production. Recently, rising incomes, a growing population, and increasing urbanization in Southeast Asia have massively increased the demand for livestock production and meat consumption, particularly poultry and pork (11). Southeast Asia’s poultry production expanded by 56% in the last decade, growing from 5.9 million metric tons (MMT) to 9.2 MMT in 2018. It is expected to reach 12.3 MMT by 2028. While the massive growth of the commercial poultry industry slowed globally during the COVID-19 pandemic, backyard poultry production increased in poorer communities and households, as it represents a viable alternative to generate income and guarantee the availability of animal protein (12). Poultry represents a particularly important source of income for Cambodian farmers (13–15). In 2015 alone, 87% of Cambodian households with agricultural holdings raised poultry, mainly on small, backyard farms (14). These figures have risen in tandem with Cambodia’s economic boom and the ongoing African swine fever (ASF) and COVID-19 syndemics (16).

Cambodia is a major hot spot for endemic and emerging infectious diseases. Avian influenza virus (AIV) is endemic in Cambodia and is a major agricultural and public health concern. Approximately 30 to 50% of ducks and 20 to 40% of chickens test positive for AIV in live-bird markets (LBMs) (17). Globally, the frequency of cross-species viral transmission is increasing (18) in tandem with a growing demand for poultry (16). This problem is inflamed by poor biosecurity practices in backyard poultry farms and markets (19). Furthermore, antibiotics are frequently used in food animal production in Cambodia to promote the well-being and growth of animals (20). Antimicrobial resistance (AMR) in bacteria found in food, animal products, and their environments is widespread and uncontrolled (21). Together, increased animal production with minimal biosecurity increases the risk of the emergence and spread of novel zoonotic diseases in the country (21).

Given that agricultural systems are integral to the livelihoods of Cambodians, it is highly feasible that tour guides who faced unemployment during the COVID-19 pandemic may have turned to backyard poultry farming to mitigate income shocks and food shortages. A pandemic-driven surge of new farmers with limited knowledge of good animal husbandry practices or standard biosecurity measures represents a significant concern for spillover and agricultural disease. Therefore, it is critical to investigate the impact of backyard farming and the potential risks of AIV and AMR in Cambodia.

## RESULTS

### Sociodemographics of respondents.

**FIG 1:**
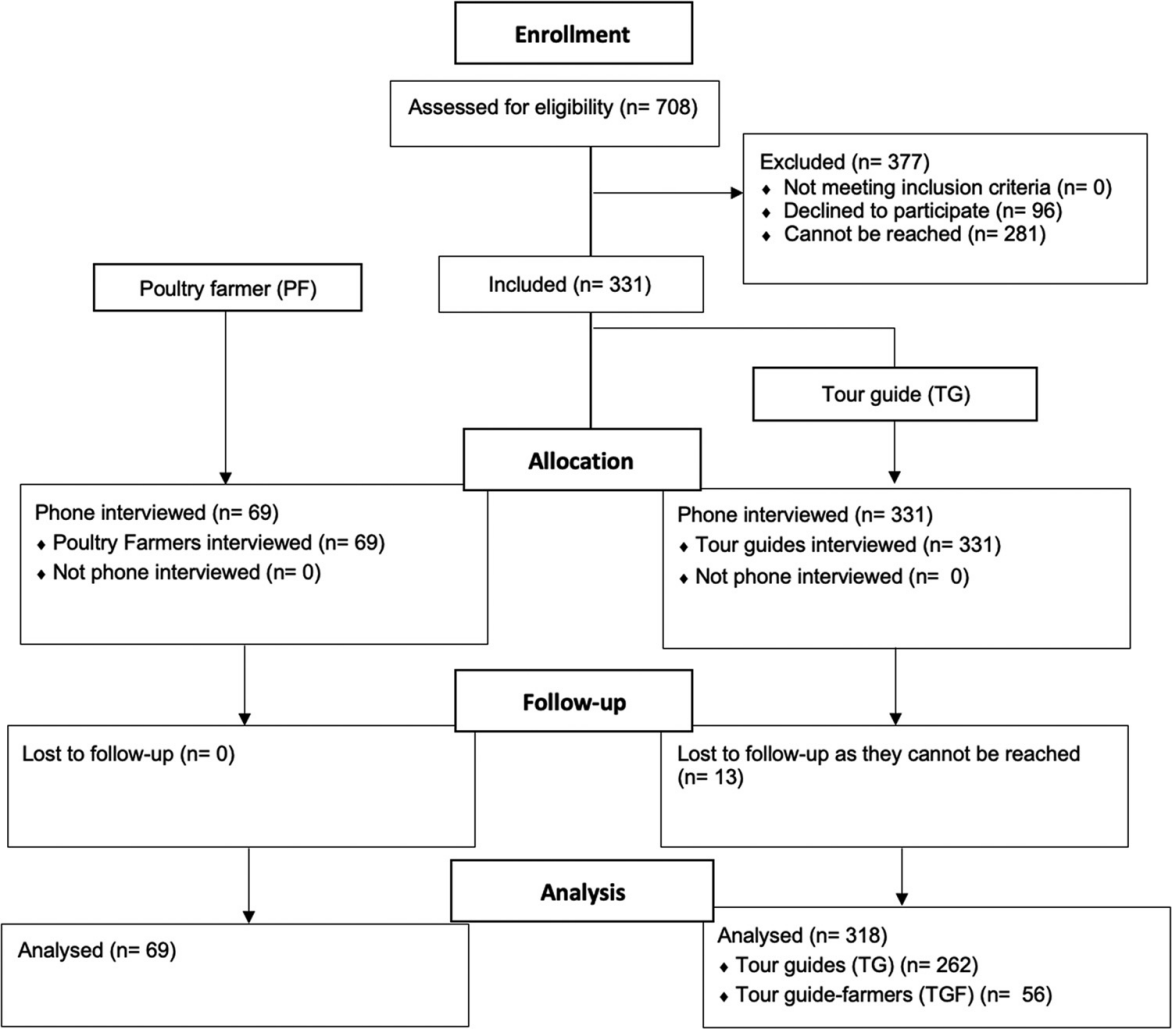
Flow diagram for the selection of study participants.

A total of 400 participants (poultry farmers [PFs], *n* = 69; tour guides [TGs], *n* = 331) were included in the study. Thirteen (4.2%) TGs were exempt from the study due to unavailability (Fig. 1). A large proportion of the respondents (88.3%; *n* = 353) were male, with an average age of 43 ± 7.77 years (Table 1). The majority (85.5%; *n* = 342) of the study respondents held a high school degree or above. Most of the respondents were household heads (85.5%; *n* = 342), with an average family size of four to seven members (73.25%; *n* = 293). The position of head of the family was largely male dominated (*P* ≤ 0.001). The majority of TGs (82.18%; *n* = 272) interviewed were from Siem Reap province; a few went back to their home provinces due to declines in working hours and job losses during the COVID-19 pandemic (Table 1).

**TABLE 1:** Demographic characteristics of survey participants

Characteristic	TGs (*n *= 331)		PFs (*n *= 69)		Total (*n* = 400)	
	No. of participants	% of participants	No. of participants	% of participants	No. of participants	% of participants
Gender						
Male	308	93.1	45	65.2	353	88.3
Female	23	6.9	24	34.9	47	11.8
Age (yrs)						
<25	1	0.3	6	8.7	7	1.8
26–36	36	10.9	12	17.4	48	12.0
36–45	187	56.5	25	36.2	212	53.0
46–55	91	27.5	19	27.5	110	27.5
>55	16	4.8	7	10.1	23	5.6
Education level						
University or above	159	48.0	6	8.7	165	41.3
Vocational/college	16	4.8	0	0.0	16	4.0
High school	149	45.0	12	17.4	161	40.3
Secondary school	6	1.8	24	34.8	30	7.5
Primary school	0	0.0	24	34.8	24	6.0
No school	1	0.3	3	4.4	4	1.0
Province at the time of the interview						
Siem Reap	272	82.2	16	23.2	288	72.0
Phnom Penh	49	14.8	0	0.0	49	12.3
Takeo	4	1.2	21	30.4	25	6.3
Prey Veng	0	0.0	12	17.4	12	3.0
Kandal	5	1.5	0	0.0	5	1.3
Kampong Cham	1	0.3	14	20.3	15	3.8
Kampong Chhang	0	0.0	4	5.8	4	1.0
Banteay Meanchey	0	0.0	2	2.9	2	0.5
Position in family						
Head	288	87.0	54	78.3	342	85.5
Spouse	12	3.6	11	15.9	23	5.8
Daughter/son	22	6.7	3	4.4	25	6.3
Mother/father	6	1.8	1	1.5	7	1.8
Other	3	0.9	0	0.0	3	0.8
Marital status						
Single	40	12.1	5	7.3	45	11.3
Married	273	82.5	60	86.9	333	83.3
Divorced	16	4.8	2	2.9	18	4.5
Widowed	2	0.6	2	2.9	4	1.0
Avg family size (no. of people)						
<3	76	23.0	15	21.7	91	22.8
4–7	240	72.5	53	76.8	293	73.3
8–11	13	3.9	1	1.5	14	3.5
>11	2	0.6	0	0.0	2	0.5

### The COVID-19 pandemic resulted in significant reductions in combined household and individual incomes, with a greater impact on tour guides than on poultry farmers.

**FIG 2:**
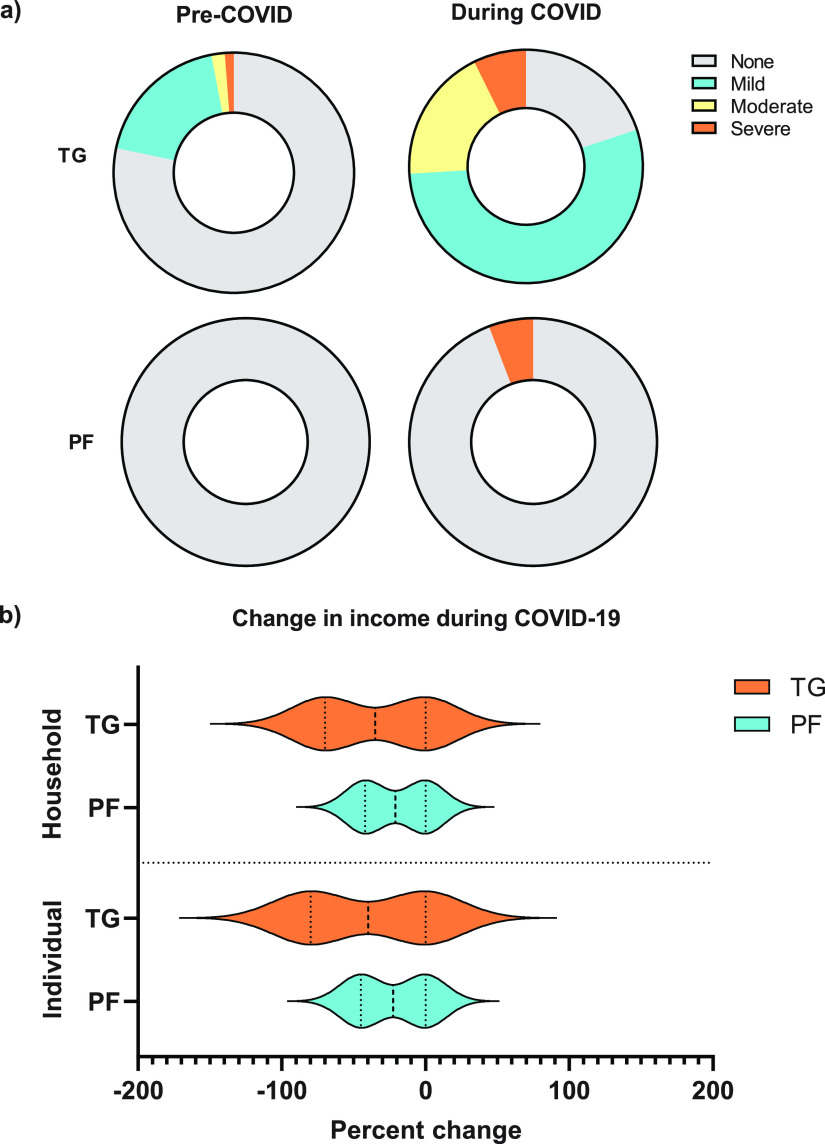
Distributions of food insecurity and income changes before versus during the COVID-19 pandemic. (a) The distributions of food insecurity based on FIES indicators are represented as categorical variables (none, mild, moderate, and severe) of the respondents’ raw scores (an integer number with a value of between 0 and 8) as the sum of affirmative responses given to the eight FIES questions. The specific categories for food insecurity are plotted by position (TG and PF). (b) Distribution of changes in income (individual and household) before and during the COVID-19 pandemic. Individual and household income data were collected as categorical variables (less than $500, $600 to $1,000, $1,000 to $2,000, and more than $2,000). Income data are graphed as percent changes.

Participants reported their incomes, before and during the COVID-19 pandemic, using categorical variables (Table 2). There were significant reductions in individual and household incomes for TGs and PFs, where TGs were more prone to extreme income contraction. Approximately 27.2% of TGs moved at least two income categories down in household income, compared to only 4.3% in the PF cohort (Fig. 2 and Table 2). Overall, monthly individual (*P* ≤ 0.05) and household (*P* ≤ 0.001) incomes significantly decreased for both TGs and PFs during the pandemic (PFs, *P* ≤ 0.001; TGs, *P* ≤ 0.001) (Table 2). Furthermore, the share of TGs earning less than $500 in individual income increased from 25% to 93.6% during the COVID-19 pandemic, while the share of PFs in the same income category increased from 36.2% to 62.3% (Table 2). Despite both groups experiencing income contraction, the PF cohort was more resilient with respect to income generation during the COVID-19 pandemic.

**TABLE 2:** Chi-squared analysis of income and food insecurity

Parameter^*a*^	Pre-COVID-19		During COVID-19		χ^2^ value (df)	*P* value
	No. of participants	% of participants	No. of participants	% of participants		
TGs						
Monthly individual income ($)*					17.2	0.008
≤500	83	25.1	310	93.7		
600–1,000	166	50.2	15	4.5		
1,000–2,000	66	19.9	6	1.8		
>2,000	16	4.8	0	0.0		
Monthly household income ($)*					34.9	<0.001
≤500	47	14.2	286	86.4		
600–1,000	144	43.5	34	10.3		
1,000–2,000	112	33.8	10	3.0		
>2,000	28	8.5	1	0.3		
Food insecurity*					69.8	<0.001
None	259	78.3	66	19.9		
Mild	62	18.7	179	54.1		
Moderate	6	1.8	62	18.7		
Severe	4	1.2	24	7.3		
PFs						
Monthly individual income ($)*					50.6	<0.001
≤500	25	36.2	43	62.3		
600–1,000	24	34.8	18	26.1		
1,000–2,000	11	15.9	8	11.6		
>2,000	9	13.0	0	0.0		
Monthly household income ($)*					49.8	<0.001
≤500	1	1.3	13	18.8		
600–1,000	34	49.3	34	49.3		
1,000–2,000	19	27.5	16	23.2		
>2,000	15	21.7	6	8.7		
Food insecurity						
None	69	100.0	65	94.2		
Mild	0	0.0	4	5.8		
Moderate	0	0.0	0	0.0		
Severe	0	0.0	0	0.0		

a * indicates that groups are significantly different (*P* < 0.05).

### Food security decreased significantly for tour guides versus poultry farmers during the pandemic.

Overall, all respondents’ food security decreased significantly during the COVID-19 pandemic (*P* ≤ 0.001). However, food security for TGs declined at a higher magnitude during the course of the pandemic than for PFs (Fig. 2). Roughly one-half (54.1%) of the TGs fell into mild food insecurity (FI), 18.7% fell into moderate food insecurity, and 7.3% fell into severe food insecurity during the COVID-19 pandemic (Fig. 2). Only 5.8% of PFs reported mild food insecurity during the COVID-19 pandemic, with the rest claiming food security (Table 2).

During the COVID-19 pandemic, the proportions of TGs concerned about not having enough food rose from 12.7% to 73.1%, not being able to eat healthy and nutritious food rose from 3.9% to 43.2%, eating a limited variety of food rose from 6.6% to 51.1%, eating less throughout the day rose from 6.6% to 50.2%, and running out of food in the house rose from 5.1% to 26% (see Table S2 in the supplemental material). In contrast, only 4.3% of PFs were concerned about not having enough food, 2.9% were unable to eat nutritious food, 1.4% ate only a few kinds of food, and none were worried about skipping a meal or running out of household food (Table S2). Several TGs took out new loans during the pandemic to support food expenses (5.12%) and household needs (14.29%), while none of the PFs acquired any type of new loan during the pandemic crisis (Table S3).

### Income and food supply disruptions led to increased interest in backyard poultry farming among tour guides during the COVID-19 pandemic.

Small-scale, backyard poultry farming represents an attractive way to address concerns about food shortages and income losses and accelerate the pace of poverty reduction (22). During the course of the COVID-19 pandemic in 2021, 69 (21%) TGs switched to small-scale backyard poultry farming to supplement food consumption. These tour guide-farmers (TGFs) were all males between the ages of 36 and 55 years, and most of them (95.65%) identified as the head of the household and the primary earner of their family (Table 3). During the COVID-19 pandemic, on average, TGFs had approximately 76 birds per farm, while PFs had 319 birds per farm. When asked about their purpose for poultry farming, 91.3% of TGFs stated that they farm for food consumption, whereas 94.2% of PFs stated that they farm for commercial purposes (Table S4). Despite the difference in coping strategies between TGFs and TGs (poultry raising versus nothing/other), there were no significant differences in demographic variables between the two groups (Table 3).

**TABLE 3:** Characteristics of tour guides who shifted to poultry farming during the COVID-19 pandemic

Characteristic	TGs (*n* = 262)		TGFs (*n* = 69)		Total (*n* = 331)	
	No. of participants	% of participants	No. of participants	% of participants	No. of participants	% of participants
Gender						
Male	239	91.2	69	100.0	308	93.1
Female	23	8.8	0	0.0	23	6.9
Age (yrs)						
<25	1	0.4	0	0.0	1	0.3
26–36	32	12.2	4	5.8	36	10.9
36–45	160	61.1	27	39.1	187	56.5
46–55	56	21.4	35	50.7	91	27.5
>55	13	4.9	3	4.4	16	4.8
Education level						
University or above	131	50.0	28	40.6	159	48.0
Vocational/college	8	3.1	8	11.6	16	4.8
High school	116	44.3	33	47.8	149	45.0
Secondary school	6	2.3	0	0.0	6	1.8
Primary school	0	0.0	0	0.0	0	0.0
No school	1	0.4	0	0.0	1	0.3
Position in family						
Head	222	84.7	66	95.7	288	87.0
Spouse	12	4.6	0	0.0	12	3.6
Daughter/son	21	8.0	1	1.5	22	6.7
Mother/father	5	1.9	1	1.5	6	1.8
Other	2	0.8	1	1.5	3	0.9
Individual income pre-COVID-19 ($)						
≤500	34	12.9	13	18.8	47	14.2
600–1,000	116	44.3	28	40.6	144	43.5
1,000–2,000	88	33.6	24	34.8	112	33.8
>2,000	24	9.2	4	5.8	28	8.5
Individual income during COVID-19 ($)						
≤500	244	93.1	66	95.7	310	93.7
600–1,000	13	4.9	2	2.9	15	4.5
1,000–2,000	5	1.9	1	1.5	6	1.8
>2,000	0	0.0	0	0.0	0	0.0
Household income pre-COVID-19 ($)						
≤500	34	12.9	13	18.8	47	14.2
600–1,000	116	44.3	28	40.6	144	43.5
1,000–2,000	88	33.6	24	34.8	112	33.8
>2,000	24	9.2	4	5.8	28	8.5
Household income during COVID-19 ($)						
≤500	222	84.7	64	92.8	286	86.4
600–1,000	32	12.2	2	2.9	34	10.3
1,000–2,000	8	3.1	2	2.9	10	3.0
>2,000	0	0.0	1	1.5	1	0.3
Food insecurity pre-COVID-19						
None	209	79.8	50	72.5	259	78.3
Mild	46	17.6	16	23.2	62	18.7
Moderate	3	1.2	3	4.4	6	1.8
Severe	4	1.5	0	0.0	4	1.2
Food insecurity during COVID-19						
None	53	20.2	13	18.8	66	19.9
Mild	145	55.3	34	49.3	179	54.1
Moderate	49	18.7	13	18.8	62	18.7
Severe	15	5.7	9	13.0	24	7.3

### The lack of biosecurity in backyard farms belonging to tour guide-farmers may act as a driver in increasing the risks of AIV infections and AMR transmission in poultry.

**FIG 3:**
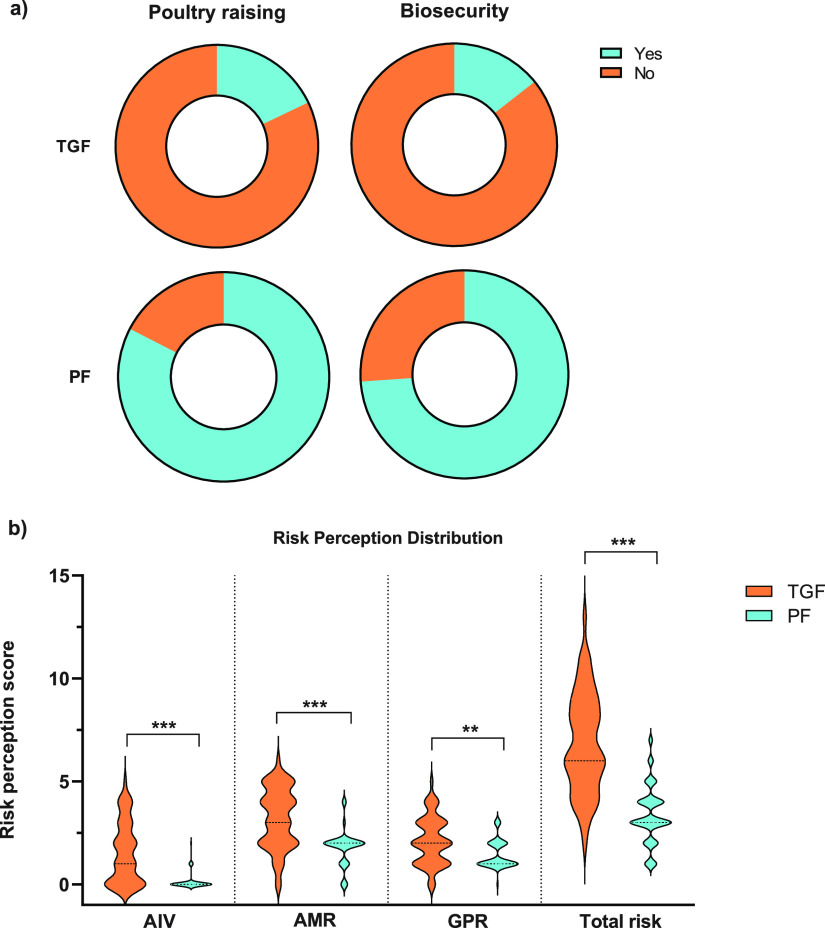
Distributions of training and risk perception scores. (a) Training attainment represented by pie charts separated by training type. Poultry raising training refers to informal and/or formal training by an experienced team on general poultry rearing. Biosecurity training refers to a period of formal education where individuals are trained in the management of poultry health and farm safety. (b) Risk perception scores of TGFs and PFs regarding AIV, AMR, GPR, and Total Risk. A higher total risk perception score is equivalent to an increased risk of AIV, AMR, and/or GPR. *, *P* < 0.05; **, *P* < 0.01; ***, *P* < 0.00.

As TGFs transitioned to small-scale backyard poultry farming, many did not receive formal training in poultry raising (82.1%) or biosecurity (85.7%) (Fig. 3). Overall, 41% of the TGFs scored higher on risk perception than PFs (*P* ≤ 0.001) (Table S5). TGFs also received less training in poultry raising (17.9%) and biosecurity training (14.3%) than PFs (Fig. 3).

The relationship between biosecurity training and the total risk perception score was not significant for the TGF cohort. However, the relationship between these two variables for PFs revealed that, on average, PFs who had received biosecurity training had a lower total risk perception score than those who had not (*P* ≤ 0.001). Furthermore, biosecurity training had a large effect (Cohen’s *d* = 0.967) on the total risk perception score, a large effect (Cohen’s *d* = 1.121) on AIV risk, no effect (Cohen’s *d* = −0.197) on AMR risk, and a large effect (Cohen’s *d* = 1.094) on general practice risk (GPR) (Cohen’s *d* = 1.094). Additionally, PFs who were trained in poultry raising scored lower in risk perception on average than those who were not (*P* ≤ 0.001) (Fig. S1).

### Biosecurity and preventive animal health services in tour guide-owned backyard farms are limited, leading to improper animal handling and an overreliance on antibiotics.

**FIG 4:**
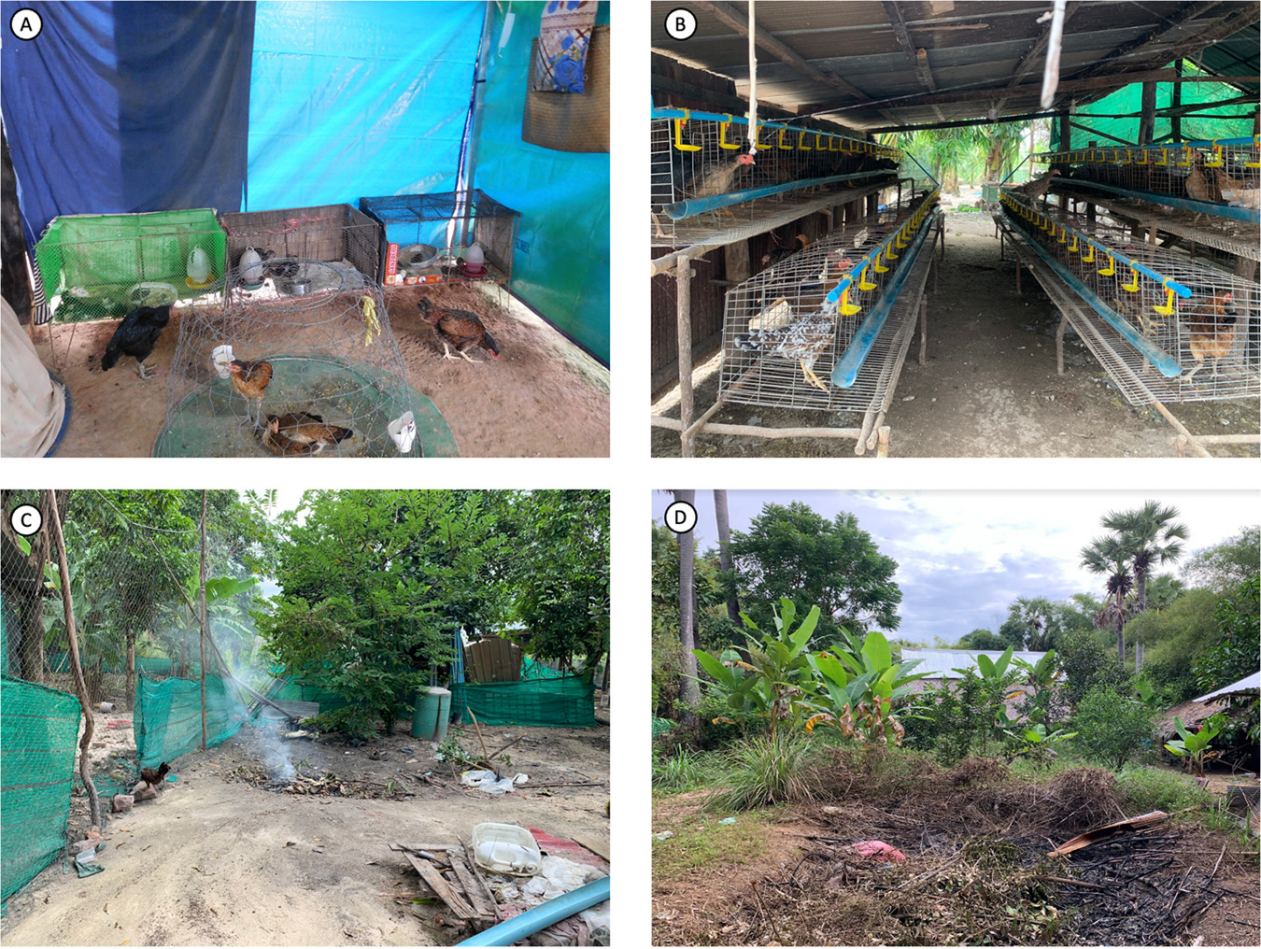
Images of tour guide-farmer (TGF) (A and C) and standard poultry farmer (PF) (B and D) poultry farms in Siem Reap province. (A) Poultry rearing arrangement of a TGF. (B) Poultry rearing arrangement of a PF. (C) Burial site for dead and/or dying poultry of a TGF. (D) Burial site for dead and/or dying poultry of a PF.

Qualitative responses from six TGFs and six PFs during the second phase of the study revealed two main themes and four subthemes. All themes and subthemes, with relevant quotations, are outlined in Table 4, and images of sample TGF and PF farms are shown in Fig. 4.

**TABLE 4:** Individual farming practices that influence AIV and AMR transmission risk in flocks

Main theme and subtheme	Subtheme definition	Relevant quote from TGF (*n* = 6)	Relevant quote from PF (*n* = 6)
Measures adopted for protecting poultry and the farmer			
Obtaining flocks from a pathogen-free source	Obtaining flocks from a producer of broiler chickens free of common poultry diseases	TGF 1: “My neighbor gave me poultry to breed and farm”	PF 5: “I get my poultry from a company near Kampong Speu province”
Treatment of sick flocks	Treating illness in poultry flocks by understanding treatment types recommended by credible personnel	TGF 5: “My friend who gave me my chickens also gave me medications, which they previously used for their sick chickens”	PF 3: “I consult with the community veterinary clinic”
		TGF 4: “I use antibiotics and medications previously that I used for my other chickens”	
Disposal of dead or dying flocks	Safe, prompt, and appropriate disposal of damaged eggs, dead birds, litter, sick or dying birds, and manure, which may carry diseases	TGF 3: “I bury all of my dead chicken carcasses outside my house, near the field”	PF 2: “I usually handle dead poultry with gloves; I burn the dead animal, and sometimes when there are too many, I bury the chickens miles from my farm”
		TGF 6: “I dig a hole in my property and bury any dead poultry there; sometimes, I will burn the dead animal first”	PF 1: “I burn the carcasses a mile away from my poultry farm”

Challenges in sustaining the farm			
Resource constraints	Self-identified economic challenges and resource constraints for maintaining sustainable backyard poultry farming during current times	TGF 4: “I recently started poultry raising, and I am still unfamiliar with making my own poultry feed, but poultry feed price increased, so it is hard to afford that now too; medicine is also sometimes expensive”	PF 4: “I had to sell at least 500 of my chickens, as feed price went 2 times higher during COVID-19, and it was hard to sustain so many chickens in my farm as there was a lack of food I could not afford”
		TGF 1: “During rainy season, many of my chickens become sick so I have to buy a lot of medication”	
		TGF 6: “I used my old restaurant’s tables and chairs to make chicken cages”	

### Measures adopted for protecting flocks and farmers.

**(i) Obtaining flocks from pathogen-free sources.** The narratives of TGFs suggest that their farming practices and poultry health management were highly influenced by information from social media and their community. TGFs reported receiving information about poultry rearing and farm management from social media, community members, and other tour guides practicing backyard farming (Table 4). When asked about the initial start of their farms, TGFs reported that they obtained their initial flock from neighbors and other TGFs. Only one TGF acquired their initial flock from a commercial farm with biosecurity measures.

**(ii) Treatment of sick flocks.** TGFs placed the most emphasis on the opinions and practices of other farmers within their communities, hence relying on other TGFs for treatment management of sick and dying poultry. One-half of the TGFs reported using miscellaneous medications, including antibiotics from other local TGFs in the community (Table 4). Conversely, all PFs reported receiving professional medical advice and treatment protocols from local community veterinary services.

Both groups reported that disease-related deaths in poultry occurred mainly during heavy rainy seasons (mid-May to early October) and during the cold season (periods between December and February). During this time, many respondents observed a higher frequency of sickness and death in their flocks and expressed concerns regarding medication affordability (Table 4). Thus, the recycling of old antibiotics was more common during these times among TGFs.

**(iii) Disposal of dead or dying flocks.** The questionnaire responses suggest that the understanding of methods of disposal of dead or dying poultry varied from person to person, with PFs following stricter biosecurity guidelines than TGFs. The majority of PFs reported burning the carcasses before burial, while many TGFs buried the carcasses directly after death. The proximities of the burial sites also differed between the two groups of farmers. PFs buried carcasses at a considerable distance from their farm locations and rearing sites (Fig. 4). TGFs discarded carcasses in proximity to their farms, in their backyards, or near empty paddy fields. Field observations from one TGF farm revealed that chickens would often roam in proximity to the burial sites (Fig. 4). The majority of PFs handled sick or dying poultry with protective gear, whereas TGFs noted the limited use of protective gear while burning carcasses (Table 4).

### Challenges in sustaining the farm: resource constraints.

Poultry feed costs were a barrier for both TGFs and PFs. Almost all participants from both groups reported a 2-fold increase in poultry feed prices compared to the prices before the COVID-19 pandemic. While this price increase resulted in many PFs downsizing their farm capacity, conversely, many TGFs sought to expand their farms.

## DISCUSSION

The COVID-19 pandemic has had a profound impact on many Cambodian households. Many families have lost employment and continue to struggle with food security (23). The present study is the first to estimate the proportion of TGs reporting experiences of lost wages and food security in their households during the COVID-19 pandemic, drawing comparisons with prepandemic levels and vis-à-vis PFs. For TGs, the most cited effect of the loss of employment relates to the cessation of tourism-related activities due to pandemic-associated travel restrictions. While both groups experienced economic hardships during the COVID-19 pandemic, poultry farming demonstrated more resilience as an income-generating strategy as the agrifood sector was designated essential and exempt from business closures. More TGs than PFs accessed new loans in an effort to mitigate the negative shocks on consumption, even though both groups reported similar access to financial services (measured as access to financial services before the pandemic).

Only TGs experienced a significant decrease in food security, likely caused by pandemic-induced disruptions in the food supply and higher food prices in the country (24). On the contrary, PFs were more resilient to food security shocks, as only a small proportion of individuals from this group reported a decrease in food security. Food security for PFs could be attributed to the duality of livestock products in poultry farming, as poultry provides both a source of nutrition and income for the farm family (25).

As hypothesized, some TGs sought to alleviate and/or supplement income losses and augment food security with small-scale, backyard poultry farming. TGFs were less concerned than PFs about the risks of AIV, AMR, and zoonotic disease spillover. Additionally, only a small proportion of TGFs were trained in biosecurity and/or poultry raising. The incursion of these new actors, characterized by their limited experience and knowledge, represents a major risk for the poultry sector in Cambodia, as limited biosecurity in backyard farms increases the risk of AIV and AMR prevalence in poultry flocks (26). Many TGFs relied on social media for information on poultry rearing, limiting standard biosecurity methods. Poultry flocks reared under limited biosecurity are three times more vulnerable to the spread of AIV than those reared on farms with adequate biosecurity (26). Thus, improving farm biosecurity measures is critical for mitigating the risk of pathogen introduction or spread (27).

While biosecurity measures have been widely adopted in the commercial poultry sector, these measures are difficult to implement and often unaffordable for backyard poultry farmers, particularly those who are new and inexperienced. In addition, being trained in biosecurity and poultry raising appears to be a major driver in decreasing risk perception in poultry farmers. These results demonstrate that PFs who were trained in biosecurity had a lower risk perception score than those who were untrained. Although the results were not significant for TGFs, likely due to the small proportion of tour guides receiving biosecurity training, there is an inverse relationship between biosecurity training and the risk perception for AIV and AMR in poultry farming (28).

One limitation of this study was that the findings were acquired via a survey method based on the recall memory of individual poultry farmers. The responses are in favor of the most digitally connected participants, and a few participants were unavailable for a follow-up survey regarding questions about their farming practices. The smaller sample size may introduce sampling bias, limiting the generalizability of the findings due to population validity. Likewise, the study does not capture a representative sample of the TG population and is most likely biased toward individuals from Siem Reap province. The temporal differences between the food insecurity experience scale (FIES) score and individual and household incomes applied before and during the pandemic may introduce temporal bias as there is a time frame gap between before and during the pandemic. Furthermore, measuring the average interactions or movements between TGF and PF farms would add another level of rigor to this study. Future research should prioritize longitudinal data collection, including bird and person movements, to strengthen the relationship between farm biosecurity and the risk of disease spillover.

As of mid- to late 2022, tourists have been returning to Cambodia as a result of the country’s strong efforts against COVID-19 (29, 30), resulting in many former tour guides returning to the tourism industry. Despite noteworthy strides toward the recovery of the tourism industry in Cambodia, 83% of the TGFs in this study reported that they expanded their backyard poultry business in parallel to resuming work as tour operators. The Department of Tourism in Siem Reap has confirmed that 58% of TGs have renewed their licenses to work as tour operators, while 42% of TGs have not (as mentioned by Ngov Sengkak, Director, and Heng Sarak, Head of Administration of the Siem Reap Provincial Department of Tourism), raising concerns regarding the types of income-generating activities of such individuals.

While poultry farming can provide resiliency to mitigate the impact of temporary income shocks, future work should focus on biosecurity educational programs to observe the association between the provision of training and the prevalence of AIV and AMR transmission in poultry. Currently, there is an organized effort in rural Cambodia to equip new farmers with resources to start safe small-scale farming. Biosecurity training and awareness campaigns for AIV and AMR should be part of this strategy. This would allow newcomers to supplement their income and reduce food insecurity without increasing the risk of zoonotic outbreaks or AMR.

### Conclusion.

While this study highlights COVID-19-associated changes in poultry production at a small scale for TGFs in Cambodia, poultry production is expected to expand due to an increase in the global demand for poultry protein during the pandemic (31, 32), changes in urbanization (33), and the reduction of the global pork supply caused by African swine fever (ASF) (34). The influx of backyard farming with minimal biosecurity is not limited to the COVID-19 pandemic, as this increased reliance is often associated with economic downturns. This global demand for and surge in poultry products, combined with inadequate biosecurity methods, can lead to an increased risk of domestic animal and human spillovers of zoonotic pathogens such as AIV. Countries in regions of endemicity are often plagued by complex emergency situations (i.e., food insecurity and economic fallouts) that hinder efforts to effectively address the emergence (or reemergence) of zoonotic diseases. Thus, novel surveillance strategies for emerging and reemerging infectious diseases should target smaller noncommercial poultry farms. It is critical to reassess and implement robust surveillance systems for peridomestic and wild animals and biosecurity training programs for backyard poultry farmers to prevent future global pandemics.

## MATERIALS AND METHODS

### Study design and participants.

To examine the implications of the COVID-19 pandemic for backyard poultry farming practices, a cross-sectional study was performed with tour guides (TGs) and poultry farmers (PFs). Potential participants were initially chosen by gathering contact information from private tourism companies, tour guide associations, and the Department of Tourism in Siem Reap province. A total of 708 tour guides were contacted for eligibility through chain referral snowball sampling. Individuals who were unreachable or refused to participate were excluded from the study during the selection-and-interview process. In total, 331 TGs participated in the study (Fig. 1). A number of PFs (*n* = 69) with small-scale backyard to mid-sized independent farms involved in other ongoing poultry/avian influenza-related research in Cambodia were selected to serve as a control group.

Power calculations were conducted to estimate the desired sample size using the share of newly food-insecure respondents as the key variable. Increases of 10% and 30% of respondents declaring being food insecure (during COVID-19) were used for the control and treatment groups, respectively. An alpha (type I error) value of 0.05 and a statistical power of 80% led to a target sample size (with balanced groups) of 124 respondents (62 for each group).

### Data collection.

Data were collected using a combination of two approaches: a standardized questionnaire and semistructured interviews focusing on sociodemographics, monthly household and individual incomes, loan attainment, the FAO’s food insecurity experience scale (FIES) (35), and modified components of a knowledge, attitude, and practice (KAP) survey regarding poultry and livestock rearing, raising, and training (36). The questions focused on the period before and during the COVID-19 pandemic (2020 to 2022) (see Appendix S1 in the supplemental material). Questionnaires were administered by trained interviewers who contacted all individuals via telephone and in-person visits.

### Ethics statement.

All respondents provided informed consent to participate in the survey. The study’s purpose was explained to all study participants before they were asked to participate in the study. Verbal informed consent was obtained from each individual via telephone before data collection. All study materials and protocols were approved by the Cambodian National Ethics Committee for Health Research (044NECHR/2022).

### Statistical analysis.

Statistical analyses were performed using Stata release 15.1 software. We used descriptive statistics (means, percentages, standard deviations [SDs], and ranges) to summarize the characteristics of the participants. The independent *t* test and the Kruskal-Wallis rank sum test were used to compare quantitative variables between categories; Cohen’s *d*, or the standardized mean difference, was used to measure the effect size between quantitative variables between categories, and the standard chi-square test was used to compare categorical variables before and during the COVID-19 pandemic. Significant differences were defined as comparisons with a 95% confidence interval (CI) exclusive of the null value of 1.0.

Food insecurity (FI) was measured using the FIES, a United Nations FAO Voices of the Hungry (FAO-VoH) experiential metric of FI (35). The total food insecurity score for each individual was calculated by adding the sum of all positive responses (yes) to the 8 FIES questions (35). Food insecurity categories (score of 0 for none, score of 1 to 4 for mild, score of 5 to 7 for moderate, and score of 8 for severe) were created based on the total sum of the scores from the eight questions.

To estimate the potential risk perception for avian influenza virus (AIV) and antimicrobial resistance (AMR) in the groups practicing small- to medium-level farming, the questionnaire was divided into three distinct modules: (i) AIV risk, including general AIV knowledge, perception, and concerns about AIV risks in poultry and the community; (ii) AMR risk, including knowledge of AMR, animal health, and poultry welfare management; and (iii) general practice risk (GPR), including biosecurity and general farm management, routine and hygienic practices, farm-to-farm contact, and slaughtering.

In each module, relevant questions were asked of study participants in two groups (Table S1). For instance, in the AIV module, emphasis was given to estimating the risk of AIV in farming. The analysis of three modules was done on the basis of a scalar scoring method. There were two types of questions requiring a binary or categorical response. Responses were assigned to specific scores to increase the generalizability of the data and the objectivity of the analysis. A score of 0 points was given for nonrisky/correct responses, and a score of 1 point was given for risky/wrong or uncertain responses. For categorical responses, scores increased by increments of 1 for every response that indicated a greater risk for the transmission indicated and/or the impacts of AIV and AMR on flocks. The total risk perception was calculated by combining the scores from the three modules. The compiled scores were then further categorized into three ranks (low, moderate, and high).

### Qualitative assessment.

Twelve respondents (six TGs and six PFs) in Siem Reap province were randomly selected for in-depth interviews about hygienic practices regarding poultry rearing and slaughter, treatment and movement of flocks, and farm hygiene practices. In-depth interviews were performed in person at each farm in Siem Reap province. Survey questionnaires were comprised of short closed-, semiclosed-, and open-ended questions in a simple, clear format to minimize confusion and maximize the response accuracy. By using a grounded theory design, qualitative responses from the participants were carefully reviewed, and emerging themes and subthemes were generated (37).

### Data availability.

The questionnaire and raw data set used or analyzed during the current study are available from the corresponding author upon reasonable request.
