# Supplementary material for: Influx of Backyard Farming with Limited Biosecurity Due to the COVID-19 Pandemic Carries an Increased Risk of Zoonotic Spillover in Cambodia
Source: Microbiol Spectr. 2022 Dec 14;11(1):e04207-22. doi: 10.1128/spectrum.04207-22 (PMC9927512; doi:10.1128/spectrum.04207-22)
Supplement: Supplemental file 1 — Supplemental material. Download spectrum.04207-22-s0001.pdf, PDF file, 1.1 MB [file spectrum.04207-22-s0001.pdf]

Supplementary Figures and Tables

1. Appendix 1: Study Questionnaire..2
2. Table S1: Risk perception (AIV, AMR, GPR) scoring index..11
3. Table S2: Specific FIES questions pre- and during COVID-19, by position...12
4. Table S3: Loan attainment pre- and during COVID-19, by position...13
5. Table S4: Motivation and/or plans for poultry products in farms, by position..14
6. Table S5: Total Risk Perception scores (AIV, AMR, and GPR) for TGF and PF....................................15
7. Figure S1: Total Risk Perception scores for TGF and PF, by biosecurity training...................................16

Appendix 1 Study Questionnaire (with Khmer language translation)

Questionnaire for new and experienced commercial/semi-intensive poultry farmers

Section 1: General Information

Address: Village:______________Commune:___________District:____________

Province: ☐ Siem Reap ☐ Phnom Penh ☐ Takeo ☐ Prey Veng ☐ Kandal

01. Respondent's name: ____________________
02. Respondent's telephone number: ____________________
03. Are you: (please tick as necessary) ☐ Male ☐ Femalee
04. Age (years): ____________________
05. Education:
    - ☐ University or above
    - ☐ Vocational/college (1-3 years)
    - ☐ High school (10-12)
    - ☐ Secondary school (6-9)
    - ☐ Primary school (1-5)
    - ☐ No school
06. How many people are living in your family? ___________________
07. Position in the family with Head of household:
    តួនាទី ក្នុងគ្រួសារជាមួយមេគ្រួសារ
    - ☐ Head
    - ☐ Spouse
    - ☐ Daughter/son
    - ☐ Mother/father
    - ☐ Other
08. Marital status:
    - ☐ Single (never married)
    - ☐ Married
    - ☐ In a domestic partnership
    - ☐ Divorced
    - ☐ Widowed
09. Occupation:
    - ☐ Tour guide
    - ☐ Farmer
    - ☐ Hospitality worker
    - ☐ Govt. officer
    - ☐ Private business
    - ☐ Free labor
    - ☐ Driver
    - ☐ Unemployed ☐ Other
10. What is your current employment status?
    តើស្ថានភាពការងារបច្ចុប្បន្នរបស់អ្នកគឺជាអ្វី?
    - ☐ Employed full time (40+ hours a week)
    - ☐ Employed part-time (less than 40 hours a week)
    - ☐ Unemployed (currently looking for work)
    - ☐ Student
    - ☐ Retired
    - ☐ Self-employed
    - ☐ Other
11. Have you received a complete dose of the COVID-19 vaccine?
    តើអ្នកបានទទួលវ៉ាក់សាំងការពារកូវីដ១៩ ហើយឬនៅ?
    - ☐ Yes
    - ☐ No

Section 2: General Information of Responder Pre-COVID-19

12. What was your monthly household income?
តើប្រាក់ចំណូលសរុបប្រចាំខែរបស់គ្រួសារអ្នកប៉ុន្មាន?
    - ☐ <=500 USD
    - ☐ 600-1000 USD
    - ☐ 1000-2000 USD
    - ☐ >=2000+ USD
13. What was your INDIVIDUAL income per month?
តើចំណូលផ្ទាល់ខ្លួនរបស់អ្នកក្នុងមួយខែប៉ុន្មាន?
    - ☐ <=500 USD
    - ☐ 600-1000 USD
    - ☐ 1000-2000 USD
    - ☐ >=2000+ USD
14. What was your main INDIVIDUAL income generating activity?
តើអ្វីជាសកម្មភាពប្រាក់ចំណូលសំខាន់របស់អ្នក?
    - ☐ Tourism operator
    - ☐ Food service and Hospitality worker
    - ☐ Agriculture
        - ☐ Poultry farming
        - ☐ Pig farming
        - ☐ Other livestock
        - ☐ Crop production
    - ☐ Other
15. How many years of experience have you had in your job?
តើអ្នកមានបទពិសោធន៍ប៉ុន្មានឆ្នាំក្នុងការងាររបស់អ្នក?
    - ☐ <1 Year
    - ☐ 1-2 Years
    - ☐ 2-5 Years
    - ☐ 5-10 Years ☐ >=10 Years
16. Did you acquire a loan or personal credit ever?
តើអ្នកទទួលបានប្រាក់កម្ចី ឬឥណទានផ្ទាល់ខ្លួនទេ?
    - ☐ Yes
    - ☐ No **if No, please go to Q18**
17. What was the main purpose for the loan?
តើអ្វីជាគោលបំណងចម្បងនៃប្រាក់កម្ចី?
    - ☐ To buy food
    - ☐ For household needs
    - ☐ Farm infrastructure
    - ☐ Livestock inputs (feed, young birds, equipment's, etc.)
    - ☐ To pay previous debts
    - ☐ Other
    - ☐ Not applicable
18. Have you actively raised or worked with livestock pre-COVID-19 (poultry, pig farming, etc.)?
តើអ្នកធ្លាប់ចិញ្ចឹម ឬធ្វើការជាមួយបសុសត្វ (បសុបក្សី ចិញ្ចឹមជ្រូក។ល។)
    - ☐ Yes
    - ☐ No **if No, please go to Q20**
    - ☐ Not applicable **if Not applicable, please go to Q20**
19. Why did you raise livestock?(Multiple answer)
ហេតុអ្វីបានជាអ្នកចិញ្ចឹមសត្វ?
    - ☐ Monetary
    - ☐ Individual consumption
    - ☐ Other
    - ☐ Not applicable

19a. How many birds were your poultry farming?

The following questions are on food insecurity experience (pre-COVID-19):
សំណួរខាងក្រោមគឺទាក់ទងនឹងបទពិសោធន៍អសន្តិសុខស្បៀង (មុនកូវីដ-១៩)៖

20. **You were worried you would not have enough food to eat?**
តើអ្នកព្រួយបារម្ភថាអ្នកនឹងមិនមានអាហារគ្រប់គ្រាន់ដើម្បីញ៉ាំទេ?
☐ Yes
☐ No

21. **You were unable to eat healthy and nutritious food?**
អ្នកមិនអាចញ៉ាំអាហារដែលមានសុខភាពល្អ និងជីវជាតិបានទេ?
☐ Yes
☐ No

22. **You ate only a few kinds of food?**
តើអ្នកញ៉ាំតែអាហារប៉ុន្មានប្រភេទទេ?
☐ Yes
☐ No

23. You had to skip a meal because you were unable to afford any food?
តើអ្នកត្រូវអត់ញ៉ាំអាហារទេ ដោយសារតែអ្នកមិនមានលទ្ធភាព?
☐ Yes
☐ No

24. You ate less than you thought you should?
អ្នកញ៉ាំតិចជាងអ្វីដែលអ្នកគិត
☐ Yes
☐ No

25. Your household ran out of food?
គ្រួសាររបស់អ្នកអស់អាហារមែនទេ?
☐ Yes
☐ No

26. You were hungry but did not eat because you could not afford any meals?
អ្នកឃ្លានតែមិនញ៉ាំ ដោយសារតែអ្នកមិនមានលទ្ធភាព?
☐ Yes
☐ No

27. You went without eating for a whole day because you could not afford any meals?
អ្នកបានទៅដោយមិនញ៉ាំអាហារពេញមួយថ្ងៃ ដោយសារតែអ្នកមិនមានលទ្ធភាព?
☐ Yes
☐ No

Section 3: Present Information of Responder (during COVID-19)

---

28. What is your monthly household income?
តើប្រាក់ចំណូលគ្រួសារប្រចាំខែរបស់អ្នកគឺជាអ្វី?
☐ <=500 USD
☐ 600-1000 USD
☐ 1000-2000 USD
☐ >=2000+ USD

29. What is your main INDIVIDUAL income generating activity?
តើសកម្មភាពប្រាក់ចំណូលផ្ទាល់ខ្លួនរបស់អ្នកគឺជាអ្វី?
☐ Tourism operator
☐ Food service and Hospitality worker
☐ Agriculture
  ☐ Poultry farming
  ☐ Pig farming
  ☐ Other livestock
  ☐ Crop production
☐ Other

30. What is your INDIVIDUAL income per month?
តើប្រាក់ចំណូលផ្ទាល់ខ្លួនរបស់អ្នកក្នុងមួយខែគឺជាអ្វី?
☐ <=500 USD
☐ 600-1000 USD
☐ 1000-2000 USD
☐ >=2000+ USD

31. How many years of experience have you had in your job?
តើអ្នកមានបទពិសោធន៍ប៉ុន្មានឆ្នាំក្នុងការងាររបស់អ្នក?
☐ <1 Year
☐ 1-2 Years
☐ 2-5 Years
☐ 5-10 Years
☐ >=10 Years

32. **Did you acquire a new loan or personal credit?**
តើអ្នកទទួលបានប្រាក់កម្ចីថ្មីឬឥណទានផ្ទាល់ខ្លួនទេ?
☐ Yes
☐ No **IF no, go to Q34**
☐ Not applicable **IF not applicable, go to Q34**

33. What is the main purpose for the loan?
តើអ្វីជាគោលបំណងសំខាន់សម្រាប់ប្រាក់កម្ចី?
☐ To buy food
☐ For household needs
☐ Farm infrastructure
☐ Livestock inputs (feed, young birds, equipment's, etc.)
☐ To pay previous debts
☐ Other
☐ Not applicable

34. Are you currently raising livestock (poultry, pigs, etc.)?
តើអ្នកកំពុងចិញ្ចឹមសត្វ (បសុបក្សី ជ្រូក។ល។)?
☐ Yes
☐ No **IF no, go to Q36 then skip Section 4, 5 and 6**
☐ Not applicable **IF no, go to Q36 then skip Section 4, 5 and 6**

34a. Did you change to poultry farming because of COVID-19?
☐ Yes
☐ No

35. Do you raise livestock for monetary reasons or was it for individual consumption?
តើអ្នកចិញ្ចឹមសត្វដោយហេតុផលរូបិយវត្ថុ ឬវាសម្រាប់ការប្រើប្រាស់ផ្ទាល់ខ្លួន?
☐ Monetary
☐ Individual consumption
☐ Other
☐ Not applicable

The following questions are on current food insecurity experience (during-COVID-19):
សំណួរខាងក្រោមនេះគឺអំពីបទពិសោធន៍អសន្តិសុខស្បៀងបច្ចុប្បន្ន (អំឡុងពេលកូវីដ -១៩)៖

36. You were worried you would not have enough food to eat?
តើអ្នកព្រួយបារម្ភថាអ្នកនឹងមិនមានអាហារគ្រប់គ្រាន់ដើម្បីញ៉ាំទេ?
☐ Yes ☐ No

37. You were unable to eat healthy and nutritious food?
អ្នកមិនអាចញ៉ាំអាហារដែលមានសុខភាពល្អ និងជីវជាតិបានទេ?
☐ Yes
☐ No

38. You ate only a few kinds of foods?
តើអ្នកញ៉ាំតែអាហារប៉ុន្មានប្រភេទទេ?
☐ Yes
☐ No

39. You had to skip a meal because you were unable to afford any food?
តើអ្នកត្រូវអត់ញ៉ាំអាហារទេ ដោយសារតែអ្នកមិនមានលទ្ធភាព?
☐ Yes
☐ No

40. You ate less than you thought you should?
អ្នកញ៉ាំតិចជាងអ្វីដែលអ្នកគិត
☐ Yes
☐ No

41. Your household ran out of food?
គ្រួសាររបស់អ្នកអស់អាហារមែនទេ?
☐ Yes
☐ No

42. You were hungry but did not eat because you could not afford any meals?
អ្នកឃ្លានតែមិនញ៉ាំ ដោយសារតែអ្នកមិនមានលទ្ធភាព?
☐ Yes
☐ No

43. You went without eating for a whole day because you could not afford any meals?
អ្នកបានទៅដោយមិនញ៉ាំអាហារពេញមួយថ្ងៃ ដោយសារតែអ្នកមិនមានលទ្ធភាព?
☐ Yes
☐ No

Section 4: Raising Poultry and Livestock's

---

44. What is your position in the farm?
តើអ្នកមានតួនាទីអ្វីនៅក្នុងកសិដ្ឋាន?
☐ Owner
☐ Employed person
☐ Owner's family
☐ Other

45. Which actions are you in charge of in the farm? (Select all that apply)
តើអ្នកទទួលខុសត្រូវលើកសិដ្ឋានមួយណា? (ជ្រើសរើសទាំងអស់ដែលអនុវត្ត)
☐ I feed the animals
☐ I administer the treatments to the sick animals
☐ I manage/supervise the farm
☐ I market the farm products ☐ Other

46. Experience- how many years do you have raising poultry?
បទពិសោធន៍- តើអ្នកចិញ្ចឹមមាន់ប៉ុន្មានឆ្នាំ?
☐ <1 Year
☐ 1-2 Years
☐ 2-5 Years
☐ 5-10 Years
☐ >=10 Years

47. Training- have you received any training on poultry raising
ការបណ្តុះបណ្តាល- តើអ្នកបានទទួលការបណ្តុះបណ្តាលលើការចិញ្ចឹមមាន់ដែរឬទេ
   a. **Ever?** ☐ Yes ☐ No
   b. **In the last 12 months?** ☐ Yes ☐ No

48. Raising livestock- what kind of livestock do you have in your farm?
ចិញ្ចឹមសត្វ- តើអ្នកចិញ្ចឹមសត្វប្រភេទណានៅក្នុងកសិដ្ឋានរបស់អ្នក?
☐ Poultry (meat)
☐ Poultry (layers)
☐ Pigs
☐ Large ruminants (cow, buffalo, etc.)
☐ Small ruminants (sheep, goats, etc.)
☐ Fish

- ☐ Other birds
- ☐ Other livestock

49. How many birds do you have in your farm? (number) |____________________|
តើអ្នកមានបក្សីប៉ុន្មានក្បាលនៅក្នុងកសិដ្ឋានរបស់អ្នក? (ចំនួន)

50. What are your plans for the poultry products coming from your farm? (Select all that apply)
តើអ្នកមានគម្រោងអ្វីខ្លះចំពោះផលិតផលបសុបក្សីដែលមកពីកសិដ្ឋានរបស់អ្នក? (ជ្រើសរើសទាំងអស់ដែលអនុវត្ត)
- ☐ Household consumption
- ☐ Marketing (to sell)
- ☐ Both
- ☐ Other

51. What percentage of your household income comes from poultry raising activities?
តើប្រាក់ចំណូលគ្រួសាររបស់អ្នកប៉ុន្មានភាគរយបានមកពីសកម្មភាពចិញ្ចឹមមាន់?
- ☐ <30%
- ☐ 30% - 50%
- ☐ 50% - 70%
- ☐ >=70% ☐ Not applicable

Section 5: General Knowledge and Attitude

---

52. Do you know you can get sick from animals?
តើអ្នកដឹងថាអ្នកអាចឈឺពីសត្វទេ?
- ☐ Yes
- ☐ No
- ☐ Somewhat

53. Do you know what Avian Influenza Virus (AIV) is?
តើអ្នកដឹងទេថា មេរោគគ្រុនផ្តាសាយបក្សី (AIV) ជាអ្វី?
- ☐ Yes
- ☐ No
- ☐ Somewhat

54. Are you concerned about future Avian Influenza Virus (AIV) in raising poultry?
តើអ្នកព្រួយបារម្ភអំពីមេរោគគ្រុនផ្តាសាយបក្សី (AIV)នាពេលអនាគតក្នុងការចិញ្ចឹមមាន់ទេ?
- ☐ Seriously concerned
- ☐ Concerned
- ☐ Slightly concerned
- ☐ Not concerned at all
- ☐ I have no opinion (Do not read to respondent until you are sure they don’t have an answer)

55. Are you familiar with antimicrobial resistance (AMR)?
តើអ្នកធ្លាប់ស្គាល់ភាពធន់នឹងមេរោគប្រឆាំងមេរោគ (AMR) ទេ?
- ☐ Yes
- ☐ No
- ☐ Somewhat

56. Do you use medicated feed?
តើអ្នកប្រើប្រាស់ថ្នាំពេទ្យដើម្បីចិញ្ចឹម?
- ☐ Yes, I always use it
- ☐ I sometimes use it
- ☐ No, I never use it
- ☐ Don’t know

56a. Did you change your practice after COVID-19?
- ☐ Yes
- ☐ No

Section 6: Concluding Questions

---

57. **Your current recovery intentions - We don’t know what pathway the COVID-19 period will take – we know the depth, but not the duration. Even so, what do you expect to do once we get into recovery? (Select all that apply)**
ការស្តារឡើងវិញ - យើងមិនដឹងថារយៈពេលកូវីដ-១៩នឹងប្រព្រឹត្តទៅដល់ពេលណាទេ - យើងដឹងពីស្ថានភាពរបស់វា ប៉ុន្តែមិនមែនរយៈពេល។ ទោះបីជាយ៉ាងនេះក្តី តើអ្នករំពឹងថានឹងធ្វើអ្វីនៅពេលស្ថានភាពប្រសើរឡើងវិញ? (ជ្រើសរើសទាំងអស់ដែលមាន)
☐ Tourism operator
☐ Food service and Hospitality worker
☐ Agriculture
  ☐ Poultry farming
  ☐ Pig farming
  ☐ Other livestock
  ☐ Crop production
☐ Other

58. Do you agree to visit your farm and provide recommendations?
តើអ្នកយល់ព្រមអោយក្រុមការងារយើងទៅមើលកសិដ្ឋានរបស់អ្នកហើយផ្តល់អនុសាសន៍ទេ?
☐ Yes
☐ No
☐ Not applicable

B. Characteristics of your poultry farm

B1. What motivated you to start/go back to poultry farming?
B2. How long did it take you to get back to/into farming?
  a. Was it precipitated by travel closure or lockdowns?
B3. What are the main challenges that you face on your farm?
B4. How do you get your birds? Could you explain the procedure you follow to get your birds?
B5. Could you explain how you sell your spent birds/finished broilers? (If individual consumption, skip)
B3. For Layers: Could you explain how do you sell your eggs and the frequency (twice a day, daily, every two days, etc.)? (If individual consumption, skip)
B4. Do you use feed that is mixed with antibiotics? (If yes, follow check-list item #7)
☐ Yes
☐ No
☐ Don’t know/Don’t answer
B5. Have you had any sudden deaths in your poultry flock lately? (If they say yes, ask what they did and if they reported)
B6. If you have ever had any other sickness or deaths in your flock, do they occur in a particular month or season?
B7. If you have sick or dead poultry, do you have direct contact with the sick/dead birds?
☐ Yes
☐ No
☐ Don’t know/Don’t answer
☐ Not applicable
B8. Did you wash your hands after touching sick/dead poultry?
☐ Yes
☐ No
☐ Don’t know/Don’t answer
☐ Not appliable
B9. If your birds have been sick, what symptoms did you observe in your flock?
☐ Feather loss (unless birds are going through a natural moult)
☐ General inactivity
☐ Discharges
☐ Abnormal droppings
☐ Dull and/or closed eyes
☐ Ruffled feathers
☐ Drooped wings
☐ Sitting on haunches or laying down
☐ Other, if so, please specify:……………………………………………..

---End section B--

C. Knowledge

1. Have you heard about bird flu?

☐ Yes
☐ No
☐ Don’t know/Don’t answer

1. Can people get bird flu by touching sick poultry?

☐ Yes
☐ No
☐ Don’t know/Don’t answer

2. Do you worry about your family/ friends getting bird flu?

☐ Yes
☐ No
☐ Don’t know/Don’t answer

3. Have you ever attended any training/communication events on biosecurity or good poultry husbandry practices?

☐ Yes
☐ No
☐ Don’t know/Don’t answer

4. Have you heard of antimicrobial resistance?

☐ Yes
☐ No
☐ Don’t know/Don’t answer

5. Have you heard of antibiotic resistance?

☐ Yes
☐ No
☐ Don’t know/Don’t answer

---End section C--

D. Attitude and practices

D1. What do you do when you find an animal of yours is sick?

D1. Have you had any sudden deaths in your poultry flock lately? (If they say yes, ask what they did and if they reported)

D2. If you have ever had any other sickness or deaths in your flock, do they occur in a particular month or season?

D3. If you have sick or dead poultry, do you have direct contact with the sick/dead birds?

☐ Yes
☐ No
☐ Don’t know/Don’t answer
☐ Not applicable

D4. Did you wash your hands after touching sick/dead poultry?

☐ Yes
☐ No
☐ Don’t know/Don’t answer
☐ Not appliable

D5. From 1 to 5, where 1 = I never examine the chickens in my farm when they get sick and 5 = I always examine the chickens in my farm when they get sick, how often do you examine the chickens closely in your farm?

I never examine my chickens 1 ☐ 2 ☐ 3☐ 4 ☐ 5 ☐ I always examine my chickens

D6. What do you do when your chickens are sick?

D7. Have you ever vaccinated your poultry? (If no, skip a)

☐ Yes
☐ No

a. Which of the following vaccines have you received and doses?

☐ Newcastle- 25 doses
☐ Newcastle- 50 doses
☐ Newcastle- 100 doses
☐ Cholera- 25 doses
☐ Cholera 50- doses

☐ Cholera 100- doses
☐ Fowl pox- 25 doses
☐ Fowl pox- 50 doses
☐ Fowl pox- 100 doses

D8. Have you ever received the following services for your poultry from Village Animal Health Workers (VAHWs) or your local Animal doctor?

☐ Vaccination
☐ Deworming
☐ Treatment
☐ Advice
☐ Other

D9. From 1 to 5, where 1 = I never give antibiotics to my chicken when they get sick and 5 = I always give antibiotics to my chicken when they get sick

I never give antibiotics 1 ☐ 2 ☐ 3☐ 4 ☐ 5 ☐ I always give antibiotics

D10. Do you consult with a veterinarian before giving antibiotics to your chicken?

☐ Yes
☐ No
☐ Don't know/Don't answer

D11. Do you give your chickens antibiotics when they are NOT sick?

☐ Yes (if yes, why.................)
☐ No
☐ Don't know/Don't answer

---End section D---

E. Closing questions

------------------------------------For former four guides only--

2. Your current recovery intentions - We don't know what pathway the COVID-19 period will take – we know the depth, but not the duration. Even so, what do you expect to do once we get into recovery/when tourism is open?
    a. Will you go back to primarily being a tour guide?
        ☐ Yes
        ☐ No
        ☐ Not sure
        ☐ Not applicable
    b. Will you continue chicken farming?
        ☐ Yes
        ☐ No
        ☐ Not sure
        ☐ Not applicable
    c. Will you continue doing both chicken farming and tour guide? If yes
        i. Do you have an idea how you will manage your farm?
        ii. Will you continue to grow the same number of animals or will you cut back?

Table S1 Risk perception (AIV, AMR, GPR) scoring index

Risk Perception Scoring Guide			
Module	Questions	Response	Score
1. Avian Influenza (AIV) risk	1. Can people get bird flu by touching sick poultry?	Yes	0
		No	+1
		Don't know	+1
	2. Have you heard about bird flu?	Yes	0
		No	+1
	3. Are you concerned about future Avian Influenza Virus (AIV) in raising poultry?	Seriously Concerned	0
		Concerned	0
		Slightly Concerned	+1
		Not Concerned	+2
	4. Do you worry about your family/ friends getting bird flu?	Yes	0
		No	+1
		Don't know	+1
2. Antimicrobial resistance (AMR) risk	1. Have you heard of antimicrobial resistance?	Yes	0
		No	+1
		Don't know	+1
	2. Do you use feed containing antibiotics for your poultry?	Yes, I always do	+3
		I sometimes do	+2
		No, I never use it	0
		Don't know	+1
	3. Do you consult with a veterinarian before giving antibiotics to your chicken?	Yes	0
		No	+1
		Don't know	+1
	4. Do you give your chickens antibiotics when they are NOT sick?	Yes	+1
		No	0
		Don't know	+1
3. General practice risk (GPR)	1. If you have sick or dead poultry, do you have direct contact with the sick/dead birds?	Yes	+1
		No	0
		Don't know	+1
	2. Did you wash your hands after touching sick/dead poultry?	Yes	0
		No	+1
		Don't know	+1
	3. Do you slaughter your chicken/other poultry on-site?	Yes	+1
		No	0
	4. How often do you examine the chickens closely in your farm?	Always	0
		Most of the time	+1
		Sometimes	+1
		Rarely	+2
		Never	+2
	5. Have you ever vaccinated your poultry?	Yes	0
		No	+1

Table S2 Specific FIES questions pre- and during COVID-19, by position

	**TG (n=331)**				**PF (n=69)**			
	**Pre-COVID-19 (n)**	**n%**	**During COVID-19 (n)**	**n%**	**Pre-COVID-19 (n)**	**n%**	**During COVID-19 (n)**	**n%**
**Q1** Worried would not have enough food to eat	42	12·7	242	73·1	0	0·0	3	4·3
**Q2** Unable to eat healthy and nutritious food	13	3·9	143	43·2	0	0·0	2	2·9
**Q3** Ate only a few kinds of foods	22	6·6	169	51·1	0	0·0	1	1·4
**Q4** Had to skip a meal	14	4·2	93	28·1	0	0·0	0	0·0
**Q5** Ate less than you thought you should	22	6·6	166	50·2	0	0·0	0	0·0
**Q6** Household ran out of food	13	3·9	85	25·7	0	0·0	0	0·0
**Q7** Hungry but did not eat	17	5·1	86	26·0	0	0·0	0	0·0
**Q8** Without eating for a whole day	9	2·7	33	10·0	0	0·0	0	0·0

Table S3 Loan attainment pre- and during COVID-19, by position

		TG (n=331)	n%	PF (n=69)	n%
**Loans**					
Pre- COVID	**Yes**	206	78·6	30	43·5
	**No**	125	47·7	39	56·5
**Main Purpose**	**Food**	10	6·1	0	0·0
	**House**	162	98·2	30	100·0
	**Farm**	10	6·1	0	0·0
	**Previous loans**	2	1·2	0	0·0
	**Other**	21	12·7	0	0·0
	**NA**	1	0·6	0	0·0
During COVID	**Yes**	64	24·4	0	0·0
	**No**	267	101·9	69	100·0

Table S4 Motivation and/or plans for poultry products in farms, by position

Question	Categorical Response	TGF (n=69)	n%	PF (n=69)	n%
Q1. Why do you raise livestock?	Monetary	36	52·2	55	79·7
	For consumption	63	91·3	53	76·8
Q2. What will you do with your poultry product?	Household	31	44·9	2	2·9
	To sell	4	5·8	6	8·7
	Both	32	46·4	61	88·4
	Other	2	2·9	0	0·0

Table S5 Total Risk Perception scores for TGF and PF in overall AIV, AMR, and GP risk in poultry

	TGF		PF		Total	
**Risk level**	*n = 56*	*%*	*n = 69*	*%*	*N = 125*	*%*
Low	3	5·4	41	59·4	44	35·2
Moderate	30	53·6	28	40·6	58	46·4
High	23	41·1	0	0·0	23	18·4

Note: Low rank (score between 0 to 4 points), Moderate rank (score between 5 to 8 points), High rank (score greater than 8 points); the Risk Perception is determined by the total score (a higher score implies higher risk).

Figure S1 Total Risk Perception scores for TGF and PF, by biosecurity training

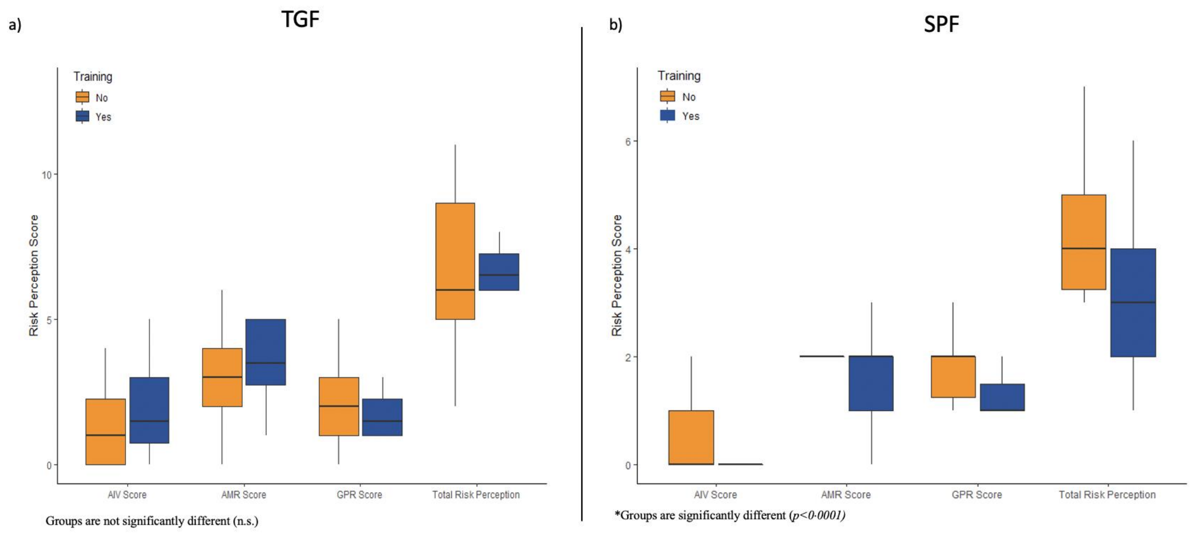

Figure S1 shows the distribution biosecurity training attainment in association to the risk perception plot showing the ranking of potential risk pathways in poultry farming among PF and TG; AIV=avian influenza, AMR= antimicrobial resistance, GPR = general practice risk, Total Risk Perception = sum of AIV score, AMR score, and GPR score. A higher risk perception score is equivalent to increased risk of AIV, and/or AMR, and/or GPR in poultry flocks between TGF and PF. Biosecurity training is shown as two categorical variables (Yes or No) in relation to the Risk Perception Score among TGF and PF. a) Left: Biosecurity training for TGF shows no significant association between biosecurity training attainment and risk perception score for AIV, AMR, GPR, and Total Risk Perception. b) Right: Biosecurity training for PF shows significant association ($p<0{\cdot}0001$) between biosecurity training attainment and risk perception score for AIV, AMR, GPR, and Total Risk Perception.
